# Porous Organic Polymers with Azo, Azoxy, and Azodioxy Linkages: Design, Synthesis, and CO_2_ Adsorption Properties

**DOI:** 10.3390/polym18060735

**Published:** 2026-03-17

**Authors:** Ivan Kodrin, Ivana Biljan

**Affiliations:** University of Zagreb Faculty of Science, Department of Chemistry, Horvatovac 102a, HR-10000 Zagreb, Croatia; ikodrin@chem.pmf.hr

**Keywords:** azo linkages, azoxy linkages, azodioxy linkages, CO_2_ adsorption, DFT calculations, GCMC simulations, porous organic polymers

## Abstract

Rising atmospheric CO_2_ levels have increased the demand for robust, scalable adsorbents for practical CO_2_ capture and separation. Porous organic polymers (POPs) are attractive candidates because their pore architecture and binding site properties can be precisely tuned via building blocks and linkage formation. This review summarizes experimental and computational studies of azo-linked POPs and, more broadly, nitrogen–nitrogen (N–N) linked systems, emphasizing how synthetic routes, building blocks, and framework topology govern CO_2_ uptake. We highlight key synthetic strategies and representative systems, including porphyrin–azo networks, and discuss the relatively sparse experimental literature on alternative N–N linked POPs incorporating azoxy and azodioxy motifs. Emphasis is placed on reversible nitroso/azodioxide chemistry as a potential pathway to ordered porous organic materials. Computational studies provide a practical route to connect structure with adsorption behavior in largely amorphous or partially ordered networks. We review hierarchical workflows combining periodic DFT and electrostatic potential properties, grand canonical Monte Carlo (GCMC) simulations, and binding energy calculations to rationalize trends and identify favorable binding environments. Computational findings demonstrate that pore accessibility and stacking models can strongly influence predicted CO_2_ adsorption. This review provides guidelines for designing POPs with enhanced CO_2_ adsorption, offering an outlook and discussing challenges for future studies.

## 1. Introduction

The continued rise in atmospheric carbon dioxide (CO_2_) levels has intensified efforts to develop new materials capable of efficient capture and separation under practical conditions [1,2,3,4]. Since pre-industrial times (~280 ppm), atmospheric CO_2_ has increased to over 427 ppm, intensifying interest in scalable adsorption-based approaches for mitigation [5,6]. A wide range of porous solids—including zeolites, activated carbons, metal–organic frameworks (MOFs), and porous organic polymers (POPs)—have been explored as CO_2_ adsorbents [7,8,9,10,11,12,13,14,15,16,17,18,19,20,21,22]. Among them, POPs offer an attractive balance of chemical and thermal stability, low skeletal density, and structural tunability, enabling pore architecture and functionality to be adjusted directly through monomer design and linkage chemistry [23,24,25,26].

For POPs, achieving high CO_2_ uptake and selectivity generally requires more than high surface area—the local chemical environment and the presence of heteroatoms often dominate adsorption strength and CO_2_/N_2_ selectivities [27,28]. Because CO_2_ has a larger quadrupole moment than N_2_, incorporating CO_2_-philic polar or Lewis-basic motifs (e.g., imines, triazines, carbazoles, and nitrogen-nitrogen type functionalities) can strengthen framework–CO_2_ interactions and improve selectivity, sometimes even when porosity is only moderate [23,24,25,26,29,30,31,32,33,34,35].

**Figure 1:**
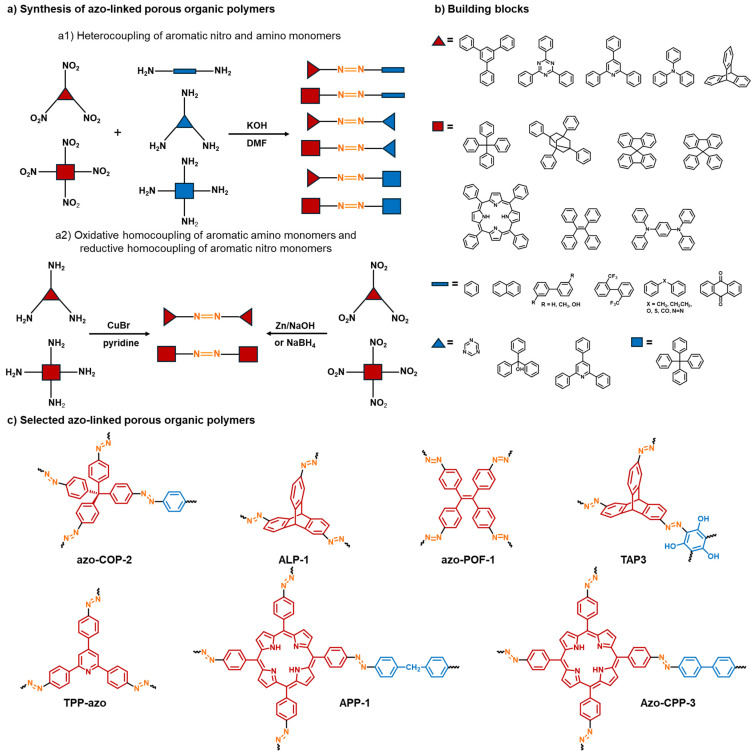
Synthesis, building blocks, and selected examples of azo-linked porous organic polymers (POPs). (**a**) Schematic representation of common synthetic approaches toward azo-linked POPs, including: (**a1**) heterocoupling of aromatic nitro and amino monomers; (**a2**) oxidative homocoupling of aromatic amino monomers and reductive homocoupling of aromatic nitro monomers. (**b**) Representative building blocks successfully employed in the synthesis of azo-linked POPs. (**c**) Structural formulas of selected azo-linked POPs.

Azo linkages (–N=N–) have drawn sustained attention because they can simultaneously increase CO_2_ affinity and, as proposed in studies, promote “N_2_-phobic” behavior that boosts CO_2_/N_2_ selectivity—an especially desirable feature for post-combustion capture [27,28]. Several robust synthetic approaches exist for building azo-linked POP networks, including catalyst-free heterocoupling of aromatic nitro and amino monomers, copper(I)-catalyzed oxidative homocoupling of aromatic amino monomers, and Zn- or NaBH_4_-mediated reductive homocoupling of aromatic nitro monomers (Figure 1a). These and other methods (e.g., diazo-coupling reactions) enable broad chemical diversity across connector geometries and pore topologies [27,36,37,38,39].

A complementary strategy to strengthen CO_2_ binding is the integration of porphyrin units, which provide rigid, π-rich platforms and basic pyrrolic environments that can serve as additional adsorption sites [40,41,42,43,44,45,46]. Combining porphyrins with azo-based linkages has therefore emerged as a promising design concept, with literature examples reporting high CO_2_ uptakes and selectivities in azo-bridged porphyrinic polymers [47,48,49].

Recent work from our group used an integrated experimental and computational approach to interpret adsorption trends in azo-linked porphyrin-based POPs, with particular focus on topology, stacking/accessibility, and local polarity [33,34].

A potentially effective strategy to enhance CO_2_ binding affinity is to move beyond non-oxygenated azo bond (–N=N–) toward oxygenated N–N linkages, i.e., azoxy (–N=N(O)–) and azodioxy (–ON=NO–) groups, which are expected to increase linkage polarity and generate more negative electrostatic regions along the pore surfaces [50,51]. Unlike azo formation—which is typically irreversible and yields amorphous POPs—azodioxy linkages form via reversible dimerization/polymerization of aromatic C-nitroso species [52,53,54,55,56,57]. Such reversibility facilitates error correction during network formation [58], potentially leading to high structural order, as exemplified by the synthesis of monocrystalline diamondoid covalent organic networks from tetrahedral polynitroso monomers [59]. Azodioxy linkages have also been incorporated into porphyrin-based crystalline covalent organic framework (COF) prepared under ambient, homogeneous solution condition [60]. Importantly, azo/azoxy/azodioxy functionalities are chemically interrelated, offering a practical way to tune local polarity without fundamentally changing the underlying connector geometry [61]. Namely, while azodioxides are formed by the dimerization or polymerization of aromatic nitroso monomers, the condensation of nitroso and *N*-hydroxylamine species yields azoxy compounds. In contrast, the condensation of nitroso and amine monomers produces azo linkages.

In our computationally assisted CO_2_ adsorption studies on the POP series built from trigonal units, periodic Density Functional Theory (DFT) and grand canonical Monte Carlo (GCMC) simulations indicated that replacing azo with azoxy, and especially azodioxy, linkages can enhance CO_2_ affinity and uptake, highlighting linkage oxidation level as a practical design variable [50,51]. In a subsequent study, we synthesized polynitroso precursors that self-associate in the solid state into *E*-azodioxy oligomers/polymers, demonstrating a practical nitroso-to-azodioxy route as an alternative pathway toward N–N linked porous networks [62].

A persistent challenge in POP research is that many materials are amorphous or only partially ordered, which limits direct structure determination and complicates the development of quantitative structure–property relationships. Computational modelling, therefore, plays a central role in interpreting trends and guiding design: binding energy calculations and electrostatic descriptors can rapidly rank functional motifs, while periodic models combined with GCMC simulations can connect pore geometry, stacking, and binding site exposure to predicted isotherms, selectivities, and adsorption thermodynamics [33,34,50,51,62,63].

This review provides a comprehensive overview of experimental and computational approaches to azo-linked and, more broadly, N–N-linked (specifically azoxy and azodioxy) POPs. We focus on how synthetic routes, building blocks, and structural order govern CO_2_ adsorption, and how modelling choices—ranging from fragment vs. periodic representations to stacking treatments and descriptor selection—shape predicted CO_2_ adsorption trends. First, we discuss progress in the synthesis and CO_2_ capture performance of azo-linked POPs, highlighting the best-performing systems. We also present the relatively limited experimental studies on azodioxy and azoxy-linked POPs and their CO_2_ uptake characteristics. Subsequently, we review computational methodologies and theoretical studies concerning the structure and adsorption features of these materials, offering a direct comparison with experimental data. The Section 5 summarizes the reported findings and outlines future directions for N–N linked POPs in advanced CO_2_ capture and catalytic conversion, with a brief perspective on emerging hybrid materials and machine learning guided design.

## 2. Synthesis and CO_2_ Capture Performance of Azo-Linked POPs

### 2.1. Synthesis of Azo-Linked POPs

Several synthetic routes toward the azo-linked POPs have been developed, incorporating diverse building blocks to fine-tune their functional (e.g., CO_2_ adsorption) properties (Figure 1). Patel et al. synthesized a series of POPs bearing azo linkages (azo-COPs) by metal catalyst-free heterocoupling of aromatic nitro and amino monomers under basic conditions (Figure 1a) [27,28]. The reaction proceeds in DMF at 150 °C for 24 h. The resulting azo-COPs exhibit various surface areas ranging from 11 to 730 m^2^ g^−1^. Another common synthesis approach to azo-linked POPs involves copper(I)-catalyzed oxidative homocoupling of aromatic amino monomers (Figure 1a). By using this method, Arab et al. synthesized highly porous azo-linked polymers (ALPs) with BET surface areas up to 1235 m^2^ g^−1^ [36,37]. They suggested that a stepwise increase in temperature and the use of mixed solvents (e.g., toluene and THF) are essential for obtaining ALPs with high surface areas. The reaction was carried out in three steps: at room temperature for 24 h, then at 60 °C for 12 h, and at 80 °C for a further 12 h. While polymerization at ambient temperature minimizes side reactions, increased catalytic activity of the catalyst at higher temperatures minimizes incomplete polymerization. Liu et al. synthesized azo-functionalized microporous organic polymers (Azo-MOPs) via oxidative polymerization of aromatic amines catalyzed by *t*-BuOCl/NaI [64]. The reaction proceeded rapidly (1 h) at room temperature, affording polymeric products with surface areas up to 706 m^2^ g^−1^ and high yields (>95%). Azo-linked POPs are often synthesized by reductive homocoupling of aromatic nitro monomers. Lu et al. reported Zn-induced reductive homocoupling of four-folded tetragonal and tetrahedral nitro-containing monomers (Figure 1a) to synthesize azo-linked porous organic frameworks (POFs) [38]. The optimized reaction conditions included the use of a mixture of THF and DMF solvents, NaOH, a temperature of 65 °C, and a reaction time of 36 h, resulting in azo-POFs with moderate BET surface areas (up to 712 m^2^ g^−1^). Reductive homocoupling of four-folded monomers containing nitro groups was much more time-efficient when using NaBH_4_ instead of Zn. Namely, NaBH_4_-mediated reductive homocoupling on polynitro monomers produced azo-linked POPs (Figure 1a) of high BET surface area (up to 1478 m^2^ g^−1^) in only 30 min [39,65]. Furthermore, azo-linked POPs containing carbazole and phenothiazine moieties, with BET surface areas up to 346 m^2^ g^−1^, were synthesized in 8 h by adapting the reductive homocoupling of the corresponding nitro monomers in the presence of NaBH_4_ [66]. Nevertheless, this method was significantly more time-consuming, with a reaction time of 24 h, when employed for the synthesis of azo-linked POPs with trigonal triphenyltriazine and triphenylpyridine central units [63]. Recently, an optimized protocol for the rapid microwave-assisted synthesis of azo-linked POPs by NaBH_4_-mediated reductive homocoupling of three and four-folded aromatic nitro monomers was reported by our group [32]. The POPs were prepared in high yields with reduced reaction times when compared to their counterparts obtained by conventional heating, especially those bearing trigonal triphenyltriazine (15 min reaction time under microwave heating vs. 24 h under conventional heating) and triphenylpyridine units (5 min reaction time under microwave heating vs. 24 h under conventional heating). Notably, polymers obtained by microwave-assisted synthesis showed similar structural, thermal, and functional characteristics as their counterparts synthesized by conventional heating. In addition to metal-catalyzed oxidative homocoupling of aromatic amines, reductive homocoupling of nitroaromatics, and the environmentally friendly condensation of aromatic amino and nitro monomers, azo-linked POPs can also be synthesized via diazo-coupling reactions. This approach was used for the synthesis of several POPs, including *o*-hydroxyazobenzene POPs [67], triptycene-based azo-linked polymers [68], POPs containing phenolic and azo moieties [69,70], azo-bridged POP bearing triazine and phloroglucinol moieties [71], and a genistein-based POP [72]. Diazo-coupling reactions between the corresponding diazonium salts and polyhydroxybenzenes were performed under mild conditions in aqueous solution, yielding POPs with BET surface areas up to 772 m^2^ g^−1^. Another approach to azo-containing POPs is based on utilizing monomer units with azo groups for the construction of polymers via Suzuki coupling [73] or Friedel–Crafts alkylation [74] reactions.

Our recent study demonstrated how the choice of synthetic routes and building blocks critically influences the porosity of the resulting azo-linked polymer materials [63]. A series of benzene and triazine-based azo-linked polymers was synthesized by heterocoupling of aromatic nitro monomers and aromatic diamines of different lengths and rigidity, by oxidative homocoupling of aromatic amino monomers, and by reductive homocoupling of aromatic nitro monomers. While all obtained polymers were isolated as amorphous materials with good thermal stability, they exhibited distinctly different BET surface areas. Specifically, triazine-based azo-linked polymers synthesized via NaBH_4_-mediated reductive homocoupling of the nitro monomer (AZO-T-P2) and copper(I)-catalyzed oxidative homocoupling of the amino monomer (AZO-T-P3) exhibited the highest BET surface areas (351 and 50.8 m^2^ g^−1^, respectively). Surprisingly, benzene and triazine-based azo-linked polymers synthesized through the heterocoupling reactions of aromatic nitro monomers and various aromatic diamines exhibited very low BET surface areas. This stands in sharp contrast to azo-COPs prepared using three-dimensional (3D) building blocks, which typically display much higher porosity [27,28].

### 2.2. Azo-Linked POPs for CO_2_ Capture

The CO_2_ uptake capacity and selectivity of POPs are heavily influenced by their surface area, pore structure, and the presence of heteroatoms (e.g., nitrogen, oxygen, sulfur, and phosphorus) [9,25]. Although a high surface area and abundant micropores are primary factors, introducing heteroatoms further improves the binding affinity towards CO_2_ via specific adsorbate–adsorbent interactions. Incorporating nitrogen atoms has been proven particularly effective in enhancing the CO_2_ capture capacity and selectivity of POPs. Specifically, nitrogen atoms act as Lewis basic sites that can interact strongly with electron-deficient CO_2_ through dipole–quadrupole interactions. This selective interaction is facilitated by the substantially larger quadrupole moment of CO_2_ compared to other common gases, such as nitrogen (N_2_). Among various nitrogen-rich porous polymers, azo-linked POPs are particularly attractive due to their high affinity to CO_2_ and high CO_2_/N_2_ selectivity, which increases with temperature, as reported by Patel [27]. This unusual behavior was attributed to the N_2_-phobicity of azo groups, originating from an entropic loss of N_2_ gas molecules upon binding, although the adsorption is enthalpically favorable. Patel et al. synthesized a family of 3D porous azo-COPs via metal catalyst-free direct coupling of aromatic nitro and amino monomers under basic conditions [27,28]. To investigate the effects of building blocks on the CO_2_ and N_2_ gas sorption characteristics, a series of monomers with varying steric hindrance, rigidity, and π-surface area was utilized (Figure 1). Azo-COPs exhibited surface areas up to 729.6 m^2^ g^−1^ and CO_2_ uptake capacity up to 1.53 mmol g^−1^ at 298 K and 1 bar. Importantly, they showed excellent CO_2_/N_2_ selectivity, ranging from 95.6 to 165.2 at 298 K and 1 bar. The authors demonstrated that increasing the π-surface area resulted in an enhanced CO_2_-philic nature of the framework, reaching a remarkable CO_2_/N_2_ selectivity of 307.7 (323 K, 1 bar). The same group investigated the effect of four different polymerization routes on the CO_2_ sorption properties of chemically similar nanoporous azobenzene polymers (NABs) [75]. They revealed that the polymerization routes have a significant influence on the pore size distribution of the NABs and, subsequently, on the temperature dependence of their CO_2_/N_2_ selectivity. Specifically, it was suggested that a pore-width maximum of 6–8 Å, along with the narrow pore-size distribution and small particle size (20–30 nm) are critical for the CO_2_/N_2_ selectivity increase with rising temperatures. Given their high CO_2_/N_2_ selectivity, azo-COPs are promising candidates for post-combustion CO_2_ capture. Nevertheless, Arab et al. argued that the relatively low surface area of azo-COPs, which results in moderate CO_2_ uptake capacities, might limit their application in industrial CO_2_ capture and separation [36]. To overcome this limitation, they developed and optimized an alternative synthetic route to azo-linked porous organic polymers (ALPs) via the oxidative homocoupling of aromatic amine building blocks, utilizing a copper(I) bromide/pyridine system. This synthetic approach yielded ALPs with significantly higher surface areas (up to 1235 m^2^ g^−1^) and superior CO_2_ storage capacities (up to 5.37 mmol g^−1^ at 273 K and 1 bar) compared to previously reported azo-COPs. However, ALPs exhibited lower CO_2_/N_2_ selectivities (ranging from 26 to 56 at 298 K and 1 bar) compared to azo-COPs [36,37]. Moreover, while azo-COPs display enhanced CO_2_/N_2_ selectivity with increasing temperature, the selectivities of ALPs decrease or remain nearly constant as the temperature increases. The authors have attributed this inconsistency to the differences in porosity parameters of ALPs and azo-COPs. It was suggested that the N_2_-phobicity in porous polymers is due to a relatively large mesopore portion, which leads to a decrease in N_2_ uptake capacity and consequently enhanced CO_2_/N_2_ selectivity values at higher temperatures [76]. Comparison of N_2_ adsorption–desorption isotherms recorded at 77 K indicated that ALPs have a lower degree of mesoporosity than azo-COPs, which show a relatively large portion of mesopores. The role of porosity parameters on the N_2_-phobicity character of azo-linked polymers was further supported by the CO_2_/N_2_ selectivities of azo-POFs, which were synthesized via Zn-induced reductive homocoupling of aromatic nitro monomers [38]. Compared to, e.g., ALP-1 (Figure 1c), which possesses a very high surface area (1235 m^2^ g^−1^) and a high CO_2_ uptake capacity (3.2 mmol g^−1^ at 298 K and 1 bar) but low CO_2_/N_2_ selectivity (28 at 298 K)**,** azo-POFs (Figure 1c) exhibit much lower surface areas (up to 710 m^2^ g^−1^) and CO_2_ uptake capacities (up to 1.9 mmol g^−1^ at 298 K and 1 bar) but yield higher CO_2_/N_2_ selectivity values (ranging from 37 to 42 at 298 K). High CO_2_/N_2_ selectivities (from 91 to 103 at 298 K) were also reported for triptycene-based azo-linked polymers (TAPs) bearing phloroglucinol units (Figure 1c) [68]. TAPs display hierarchical porosity, characterized by the coexistence of micropores and mesopores, exhibiting moderate surface areas (up to 772 m^2^ g^−1^) and high CO_2_ uptakes (up to 150 mg g^−1^ at 273 K and 1 bar). Another azo-linked POP with an incorporated triptycene moiety (Azo-Trip) was synthesized via Zn-induced reductive homocoupling of the corresponding nitro monomer [77]. Azo-Trip exhibits a somewhat lower surface area (510.4 m^2^ g^−1^), CO_2_ uptake capacity (119.6 mg g^−1^ at 273 K and 1 bar), and CO_2_/N_2_ selectivity (38.6 at 273 K) relative to the best-performing TAPs. Furthermore, a triptycene-based hypercrosslinked porous polymer (TBHCP-OH), obtained via Friedel-Crafts alkylation, has a lower BET surface (234.9 m^2^ g^−1^) and a CO_2_ capture capacity (87.6 mg g^−1^ at 273 K and 1 bar) compared to TAPs and Azo-Trip [74]. Overall, the observed differences in CO_2_/N_2_ selectivity and the N_2_-phobicity of POPs containing similar building blocks indicate that the pore architecture exerts a significant influence on their adsorption behavior [37,76,78].

**Figure 2:**
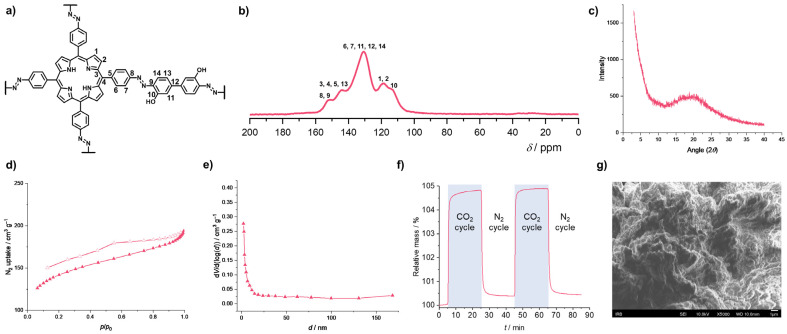
Azo-linked porous organic polymer with hydroxylated biphenyl linker (APP-BP-OH). (**a**) Molecular structure with atom numbering. (**b**) ^13^C CP/MAS NMR spectrum. (**c**) PXRD pattern. (**d**) N_2_ adsorption–desorption isotherms measured at 77 K. Adsorption and desorption isotherms are depicted with filled and empty markers, respectively. (**e**) Pore size distribution. (**f**) Thermogravimetric CO_2_ adsorption and desorption profiles. (**g**) Scanning electron microscopy (SEM) image. Reprinted with permission of [34]. Copyright 2026, American Chemical Society.

In our recent studies, we investigated the structural and functional properties of azo-linked POPs featuring various trigonal (e.g., triphenylbenzene, triphenyltriazine, and triphenylpyridine) and tetragonal (tetraphenylporphyrin) building blocks. The pyridine-based azo-linked polymer (TPP-azo) (Figure 1c), synthesized via the copper(I)-catalyzed oxidative homocoupling of 2,4,6-tris(4-aminophenyl)pyridine (TAPP), exhibited a significant BET surface area of 606 m^2^ g^−1^ and a CO_2_ uptake of 32 mg g^−1^ (at 303 K) [51]. In another study, a series of azo-linked porphyrin-based POPs (APPs), incorporating linear, bent, and trigonal linkers between the tetraphenylporphyrin units (APP-1 to APP-6), was synthesized via the direct heterocoupling of 5,10,15,20-tetrakis(4-nitrophenyl)-21H,23H-porphyrin (TNPPR) and various aromatic diamines and triamines [33]. Furthermore, oxidative homocoupling of 5,10,15,20-tetrakis(4-aminophenyl)-21H,23H-porphyrin (TAPPR) and reductive homocoupling of TNPPR and TNPPR-Zn were used for the synthesis of APP-7a, APP-7b, and APP-8, respectively, with directly connected tetraphenylporphyrin units. A comparison of the BET surface areas of the synthesized APPs emphasized the influence of the synthetic method and building blocks on the porosity of the final polymer materials. Namely, APP-1 (Figure 1c) to APP-6 bearing linkers between the tetraphenylporphyrin moieties exhibit much higher BET surface areas (from 469 to 608 m^2^ g^−1^) compared to APP-7a, APP-7b, and APP-8 containing tetraphenylporphyrin units directly connected through azo bonds (from 0.3 to 23 m^2^ g^−1^). The BET surface areas of APP-1 to APP-6 are comparable to the values previously reported for similar azo-linked porphyrin-based POPs synthesized by the same heterocoupling method [45,46,47,48,49,79]. In general, greater surface areas resulted in higher CO_2_ capture capacities at 306 K, particularly for APP-1 (41 mg g^−1^) and APP-2 (38 mg g^−1^), which feature a flexible methylene and ethylene bridge, respectively, and APP-5 (38 mg g^−1^), which contains a nitrogen-rich melamine linker. Nevertheless, the variations in CO_2_ uptake among the APPs with and without linkers between the tetraphenylporphyrin moieties are notably less pronounced than the differences in their BET surface areas. This suggests that the presence of CO_2_-philic azo groups and pyrrole rings contributes significantly to the CO_2_ binding affinity of the APPs. The CO_2_ capture capacities of the APPs are comparable to those measured for analogous azo-linked porphyrin-based POPs featuring linkers between the tetraphenylporphyrin units, such as Azo-CPPs (39.9–55.4 mg g^−1^ at 303 K) [48], Azo-Por-Bpy-POP and Azo-Por-Dadp-POP (39.1 and 36.8 mg g^−1^, respectively, at 298 K) [79]. For Azo-CPPs, the calculated CO_2_/N_2_ selectivities ranged from 27.4 to 53.9 at 273 K, and from 31.2 to 107.8 at 303 K. These values are lower than those reported for Azo-COPs (63–124 at 273 K). Similarly to the Azo-COPs, the CO_2_/N_2_ selectivity of Azo-CPPs increases at higher temperatures, highlighting these azo-linked porphyrin-based polymers as potential candidates for post-combustion CO_2_ capture and sequestration technologies. Other reported porphyrin-based POPs containing azo linkages show excellent CO_2_ uptake capacities (up to 3.98 mmol^−1^ at 273 K) and high CO_2_/N_2_ selectivity (up to 64.3 at 273 K) [45,46,47]. In our most recent work, we investigated the influence of heteroatom-containing linkers—specifically hydroxylated biphenyl (APP-BP-OH) and carbonyl-bearing anthraquinone (APP-AQ)—as well as sterically hindered linkers (methylated biphenyl and phenyl in APP-BP-Me and APP-Ph-Me, respectively) on the CO_2_ capture performance of APPs [34]. Although BET surface areas of these APPs vary widely from 167 to 673 m^2^ g^−1^, the variations in CO_2_ uptake capacity remain notably less pronounced. Specifically, APP-BP-OH (Figure 2) exhibited the highest CO_2_ uptake of 49 mg g^−1^ (at 303 K), while the remaining APPs yielded comparable values of 40 mg g^−1^ (APP-AQ) and 41 mg g^−1^ (APP-BP-Me and APP-Ph-Me). These results demonstrate that CO_2_ adsorption is governed by a complex interplay of factors, including the local chemical environment and linker topology, rather than surface area alone. Beyond the influence of the nitrogen-rich porphyrin and azo units, the presence of polar hydroxyl groups facilitates additional favorable interactions between CO_2_ and the framework. This is evident from a direct comparison of CO_2_ uptakes: APP-BP-OH achieves 49 mg g^−1^ (at 303 K), whereas its counterpart with an unsubstituted biphenyl linker, Azo-CPP-3 (Figure 1c), reaches 39.9 mg g^−1^ (at 303 K) [48]. Furthermore, the biphenyl linker itself appears to enhance CO_2_ capture capacity regardless of the surface area. Despite APP-BP-Me having a significantly lower BET surface area (167 m^2^ g^−1^) than APP-Ph-Me (673 m^2^ g^−1^), both materials exhibit identical CO_2_ uptakes of 41 mg g^−1^.

In general, the CO_2_ uptake capacities of azo-linked POPs are lower than those of top-tier metal–organic frameworks (MOFs), such as Mg-MOF-74 (8.6 mmol g^−1^ at 298 K and 1 bar) [80,81], CALF-20 (4.72 mmol g^−1^ at 298 K and 1 bar) [82,83], and UTSA-16 (4.18 mmol g^−1^ at 298 K and 1 bar) [84,85], as well as zeolites like Zeolite 13X (4.7 mmol g^−1^ at 303 K and 1 bar) [86] or amine-functionalized adsorbents such as PEI@HP20 (4.35 mmol g^−1^ at 298 K and 1 bar) [87]. Nonetheless, they offer several practical advantages crucial for post-combustion capture from humid flue gases, including high chemical and thermal stability, as well as superior moisture resistance, which remains a critical limitation for many other state-of-the-art adsorbents.

## 3. Synthesis and CO_2_ Capture Performance of Azodioxy and Azoxy-Linked POPs

**Figure 3:**
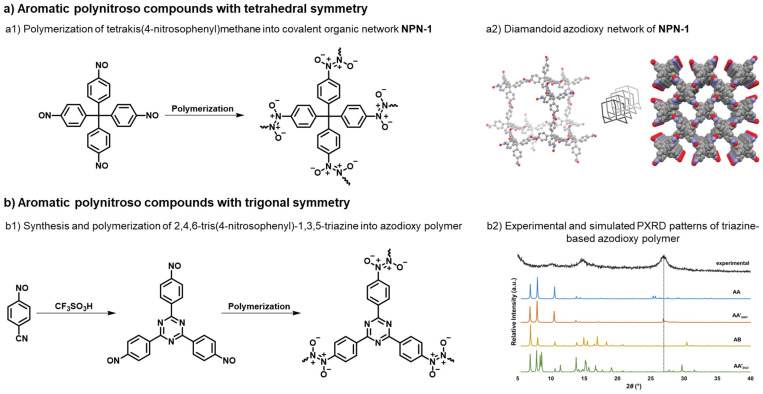
Polymerization of aromatic polynitroso compounds into azodioxy networks. (**a**) Aromatic polynitroso compounds with tetrahedral symmetry. (**a1**) Polymerization of tetrakis(4-nitrosophenylmethane) into covalent network NPN-1. (**a2**) Representation of the structure of crystals of NPN-1 showing part of the diamondoid framework (ball-and-stick image at left), the degree of interpenetration (greyscale image in the middle), and the cross-sections of parallel channels viewed along the c-axis (space-filling image at right). Atoms of carbon are shown in grey, nitrogen in blue, oxygen in red, and silicon in yellow. Guests are disordered and omitted for clarity. Adapted from [52]. Copyright 2016, American Chemical Society. (**b**) Aromatic polynitroso compounds with trigonal symmetry. (**b1**) Synthesis and polymerization of 2,4,6-tris(4-nitrosophenyl)-1,3,5-triazine into azodioxy polymer. (**b2**) Comparison of experimental and simulated (eclipsed AA, serrated AA’, staggered AB, and inclined AA’) PXRD patterns of azodioxy polymer. A peak at 2*θ* = 27.0^°^ is marked by the grey dotted line.

The drawback of azo-linked POPs is their amorphous nature, which stems from the irreversible formation of azo bonds. This structural disorder leads to several limitations in applications of these materials, such as buried surface area where pores are either too narrow or physically blocked for CO_2_ access; a broad pore size distribution that permits the co-adsorption of N_2_ and CO_2_, thereby reducing CO_2_/N_2_ selectivity; and diminished reproducibility, as the synthesis is governed by kinetic factors rather than thermodynamic equilibrium. The formation of crystalline POPs, such as covalent organic frameworks (COFs), is guided by the principles of reticular chemistry. This process requires reversible bond cleavage and formation, which allows the system to correct structural errors and reach the most thermodynamically stable, ordered state. Aromatic C-nitroso compounds are well known for their ability to reversibly dimerize or polymerize via *Z*- or, more commonly, *E*-azodioxy linkages, presenting a promising avenue within dynamic covalent chemistry [52,53,88,89]. Indeed, Beaudoin et al. showed that the self-addition polymerization of suitably designed aromatic polynitroso monomers with tetrahedrally oriented nitroso groups resulted in the formation of large single crystals of diamondoid azodioxy networks (Figure 3a) [59]. These monocrystalline covalent organic networks typically crystallize as solvates, where the solvent molecules occupy the voids. Upon the removal of these guests, the resulting solids lose their crystallinity and do not exhibit permanent porosity. Spontaneous formation of azodioxy linkages was also employed for the synthesis of a porous, semiconductive porphyrin-based COF with a reported BET surface area of 447 m^2^ g^−1^, showing I_2_-doping enhanced photoconductivity [60]. To the best of our knowledge, these rare examples of azodioxy-linked POPs have not been investigated as CO_2_ adsorbents. To fill this gap, we recently synthesized novel aromatic polynitroso compounds with *para*-nitroso groups attached to the central triphenylbenzene, triphenylpyridine, triphenyltriazine, and triphenylamine cores, and investigated their polymerization into the corresponding azodioxy networks as well as their CO_2_ capture performance [62]. The synthesis of the pyridine and amine-based derivatives was performed via a standard procedure involving the reduction of aromatic nitro derivatives to *N*-arylhydroxylamines, followed by an oxidation step yielding 2,4,6-tris(4-nitrosophenyl)pyridine and the dinitroso derivative 4,4′-((4-nitrophenyl)methylene)bis(nitrosobenzene). Since attempts to synthesize benzene and triazine-based trinitroso compounds by a similar procedure proved unsuccessful, a novel synthetic strategy was implemented, involving the cyclotrimerization of 4-nitrosoacetophenone and 4-nitrosobenzonitrile, respectively (Figure 3b). Trinitroso derivatives containing triphenylbenzene and triphenyltriazine cores underwent self-polymerization to form *E*-azodioxy linked polymers (Figure 3b), whereas pyridine-based trinitroso and amine-based dinitroso compounds formed only *E*-oligomers in the solid state. The resulting materials exhibited modest BET surface areas (up to 48.1 m^2^ g^−1^) and relatively low CO_2_ uptake values (up to 6.2 mg g^−1^ at 303 K). For comparison, analogous POPs with azo linkages, such as triphenyltriazine-based AZO-T-P2 and triphenylpyridine-based TPP-azo, show significantly higher BET surface areas of 351 m^2^ g^−1^ [63] and 606 m^2^ g^−1^ [51], respectively, as well as CO_2_ uptake capacities of 22 mg g^−1^ (at 306 K) [32] and 32 mg g^−1^ (at 303 K) [51].

During the synthesis of 4-nitrosobenzonitrile, a starting material for 2,4,6-tris(4-nitrosophenyl)-1,3,5-triazine, a nitrile-substituted azoxybenzene derivative, 1,2-bis(4-cyanophenyl)diazene oxide, was formed as a by-product. This compound was subsequently subjected to a trifluoromethanesulfonic acid-catalyzed trimerization reaction under microwave conditions to yield an azoxy-linked triazine-based polymer [90]. The resulting polymer is an amorphous solid with good thermal stability and is characterized as a wide-bandgap semiconductor. However, it exhibits a rather low BET surface area of 10.2 m^2^ g^−1^ and, in contrast to its azo and azodioxy counterparts [32,62], it shows no affinity for CO_2_ capture. To the best of our knowledge, there are no other reports on azoxy-linked POPs.

## 4. Computational Studies of N–N-Linked POPs

### 4.1. Computational Methodology for Studying Gas Adsorption in N–N-Linked POPs

Since azo-linked POPs are amorphous solids, structural information is typically limited to the expected connectivity (formation of –N=N– linkages), which was confirmed by IR and NMR spectroscopy. However, spectroscopic data alone provide little insight into how building units are arranged in 3D space. For computational modelling, certain assumptions regarding the 3D organization and topology of the material are required, as adsorption properties strongly depend on the underlying framework geometry. Therefore, idealized periodic models are often constructed to represent the connectivity between the building units and the stacking arrangements of the layers. These models enable a systematic investigation of how linkage type and layer organization influence the adsorption properties of POPs. Although not ideal, such a computational approach can reproduce the experimentally observed trends reasonably well, supporting its use as a predictive tool when experimental findings are limited.

**Figure 4:**
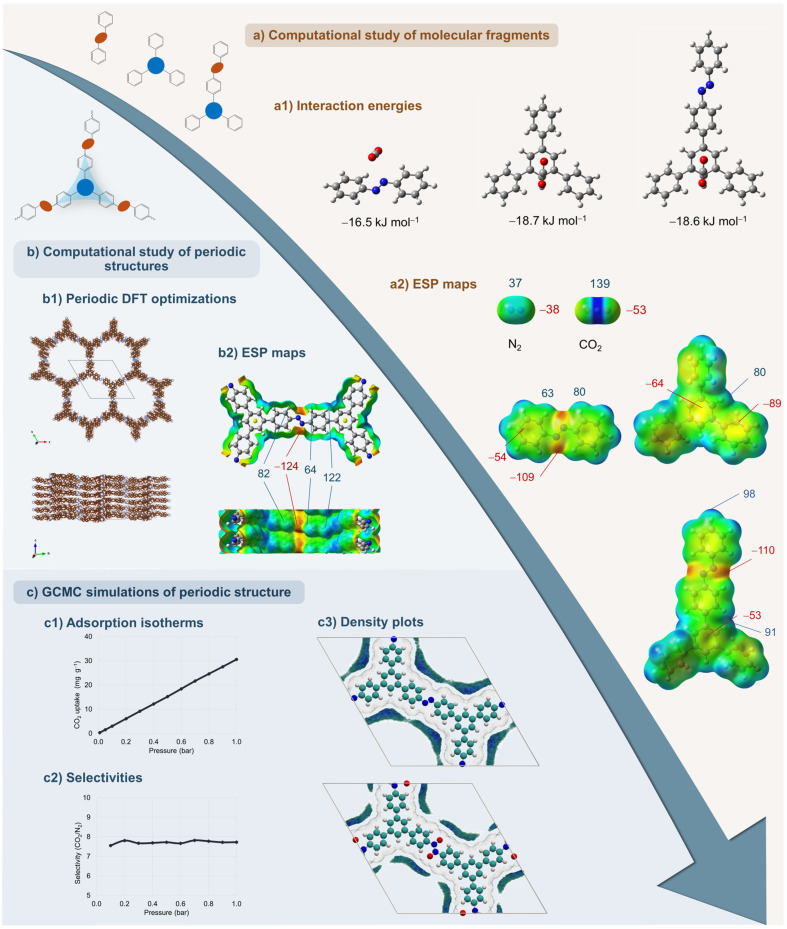
Hierarchical computational workflow for the investigation of porous organic polymers (POPs) with azo, azoxy, and azodioxy linkages. (**a**) Molecular fragment-based calculations performed on isolated molecular building blocks, including the evaluation of interaction energies with gas molecules (**a1**) and electrostatic potential (ESP) maps used to identify the most favorable gas binding sites (**a2**). (**b**) Periodic DFT calculations of the extended framework, including geometry optimizations of 3D periodic structures (**b1**) and ESP maps providing insight into the local chemical environment (**b2**). (**c**) GCMC simulations of gas adsorption in the periodic frameworks, resulting in adsorption isotherms (**c1**), selectivities (**c2**), and density distribution of adsorbate inside the pores (**c3**). Depending on the investigated system, computational studies of molecular fragments can be performed independently, particularly when the 3D periodic structure is unknown or not experimentally resolved but can be predicted from the synthetic protocol. Adapted from [50]. Copyright 2022, Royal Society of Chemistry.

The computational methodology used throughout our studies follows a hierarchical strategy (Figure 4). When an experimentally resolved 3D structure was available—or when the material was closely analogous to previously characterized COFs, such as those constructed with imine linkages—the initial steps of the workflow could be omitted. Where no reliable periodic structure existed, or when the connectivity pattern was uncertain, the analysis began with the simplest level: isolated 0D molecular fragments (Figure 4a). These fragments were used to initially check binding preferences, electrostatic features of the building blocks, functional groups, and N–N linkages.

The next stage involved assembling the fragments into idealized 2D layers that captured the expected connectivity of the building blocks, such as azo bonds, but also azoxy and azodioxy linkages. Finally, these 2D layers were arranged into different plausible stacking modes to generate full 3D periodic models of the POPs (Figure 4b). This multilevel procedure allowed us to investigate the influence of connectivity, linkage type, and layer stacking even when direct structural information was unavailable, while maintaining the structural consistency of the investigated POPs.

Periodic DFT calculations are usually performed on idealized frameworks constructed from trigonal building blocks (e.g., triphenylbenzene, triphenylpyridine, triphenyltriazine, and triphenylamine) [50,51,62,63], as well as tetragonal ones (e.g., tetraphenylporphyrin) [33,34], linked via nitrogen-nitrogen bonds. Several stacking arrangements (e.g., eclipsed AA, inclined AA′, serrated AA′, and staggered AB) were optimized using the PBE-D3 functional and pob-TZVP-rev2 basis sets in CRYSTAL23 [91]. Although these models represent perfectly ordered 3D architectures, they allow for a direct comparison of the relative stabilities and pore geometries of the corresponding POPs.

GCMC simulations in RASPA [92] are the final step that is performed on the optimized periodic structures to estimate CO_2_ and N_2_ adsorption capacities, as well as CO_2_/N_2_ selectivities, under experimentally relevant conditions (Figure 4c). In addition to adsorption isotherms, simulations generate 3D adsorption density plots that show the time-averaged spatial distribution of adsorbate molecules, providing direct insight into the preferred adsorption sites for gas molecules within the pores. Furthermore, GCMC simulations with a single adsorbate molecule in the framework enable the calculation of adsorption enthalpies, typically evaluated at the infinite dilution, where adsorbate–adsorbate interactions are negligible.

In combination with ESP maps derived from periodic DFT, the simulations show that the most negative ESP regions of the framework closely correspond to the preferred CO_2_ adsorption sites. Together, periodic DFT, ESP analysis, and GCMC simulations provide a practical hierarchical approach for interpreting the adsorption behavior of POPs. These periodic models should, however, be regarded as idealized reference structures rather than exact structural representations of the experimentally obtained materials. Their main purpose is to isolate the relative effects of linkage chemistry, connector topology, electrostatic environment, and interlayer displacement on pore accessibility and adsorption behavior under controlled structural conditions. Because real POPs may contain substantial disorder, defects, partial pore blocking, or incomplete network formation, the calculated surface areas, adsorption sites, and gas uptakes are interpreted primarily on a semi-quantitative basis. Where possible, the models are cross-checked against experimental observables, including adsorption trends and PXRD simulations used to assess plausible stacking arrangements. This hierarchical approach is particularly useful when crystallographic information is lacking or limited, as it still provides meaningful insight into the structural factors governing adsorption.

### 4.2. Computational Studies of Azo-Linked Frameworks for CO_2_ Adsorption

Computational studies on azo-linked frameworks in the specific context of CO_2_ adsorption remain relatively scarce. Most published modelling has instead emphasized electronic structure and stacking stability or disorder, whereas adsorption thermodynamics and quantitative structure-property relationships (e.g., pore accessibility vs. binding-site polarity vs. stacking) are still comparatively less explored.

In our studies, computational analysis of azo-based frameworks primarily served to clarify how the intrinsic properties of the azo linkage, in combination with connector geometry and layer stacking (Figure 4a), influence CO_2_ adsorption (Table 1). Azo linkages in individual fragments showed the weakest interaction with CO_2_ among the three types of N–N linkages investigated (i.e., azo, azoxy, and azodioxy). Azo-linked benzene and triazine frameworks were modelled in their energetically preferred AA (eclipsed) and the less stable AB (staggered) configurations. For benzene-based azo systems, CO_2_ uptakes obtained from GCMC simulations increased from 19 mg g^−1^ (AA) to 65 mg g^−1^ (AB), demonstrating that interlayer displacement can expose additional binding regions and enhance CO_2_ uptake. In triazine-based azo systems, this effect was even more pronounced, and the more accessible triazine units in AB stacking increased the predicted CO_2_ uptake from 14 mg g^−1^ (AA) to 92 mg g^−1^ (AB) [50].

**Figure 5:**
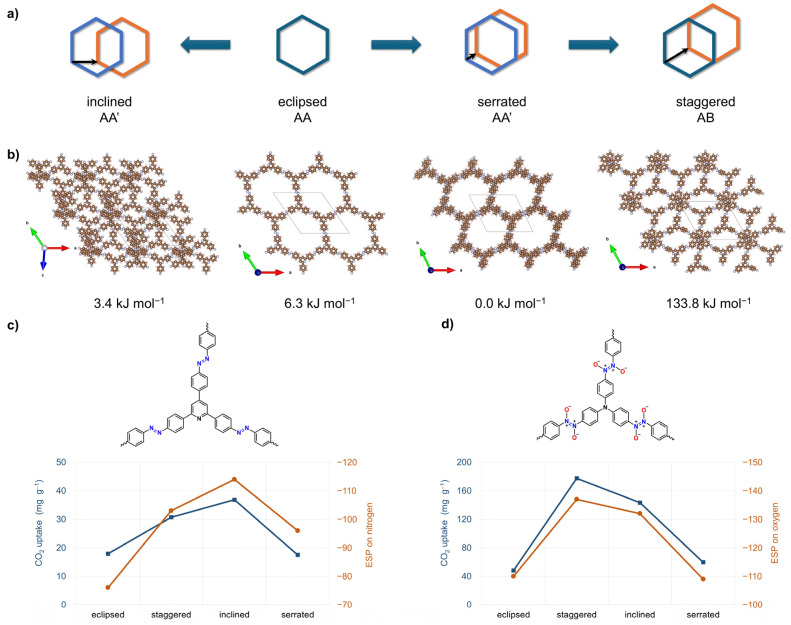
Effect of interlayer stacking on CO_2_ adsorption in layered azo- and azodioxy-linked porous organic polymers. (**a**) Schematic representation of four idealized stacking modes between adjacent 2D layers (blue and orange hexagons): inclined, eclipsed, serrated, and staggered. Black arrows indicate the direction of relative in-plane layer displacement. (**b**) Optimized periodic structures highlighting how the stacking mode changes pore shape and size, and the local chemical environment in the azo-linked polymers derived from 2,4,6-tris(4-substituted phenyl)pyridine (TPP). Crystallographic *a*-, *b*-, and *c*-axes shown in red, green, and blue colors, respectively. (**c**) Structure-property correlation between ESP values at azo nitrogen atoms and calculated CO_2_ uptakes for the four stacking modes of the azo-linked pyridine-based connectors (TPP). (**d**) Structure-property correlation between ESP values at azodioxy oxygen atoms and calculated CO_2_ uptakes for the four stacking modes of the azodioxy-linked amine-based connectors (TPA). Adapted from [51]. Copyright 2023, Royal Society of Chemistry.

The study expanded the analysis to triphenylamine and triphenylpyridine azo frameworks and further introduced additional stacking modes, including inclined AA′ and serrated AA′ arrangements (Figure 5b) [51]. In the case of triphenylpyridine, the relative energies of these geometries lie within a relatively small energy window of about 7 kJ mol^−1^, indicating the coexistence of differently stacked configurations. For triphenylpyridine, the CO_2_ uptake shows less pronounced variations compared to other investigated frameworks, ranging from 18 mg g^−1^ for the AA and serrated stackings to 31 mg g^−1^ in the AB mode, and reaching 37 mg g^−1^ for the inclined configuration. For this compound, an excellent correlation was observed between the negative electrostatic potential values on the framework surface, particularly around the azo nitrogen atoms, and the simulated CO_2_ uptake. This supports the use of ESP as an informative qualitative descriptor for identifying the most favorable adsorption configurations in azo-linked systems (Figure 5c). These findings also provided a useful basis for subsequent comparison with azoxy and azodioxy analogues, where the introduction of oxygen substantially increases linkage polarity and influences CO_2_ binding affinity [51].

The computational study was extended to a series of azo-linked POPs constructed from trigonal benzene and triazine connectors combined with linear linkers of varying length, which expand the predicted hexagonal pores via insertion between the building blocks [63]. Idealized periodic models of azo-linked benzene and triazine-based frameworks were constructed and optimized using periodic DFT, assuming predominantly eclipsed AA stacking as a preferred reference structure. The calculated CO_2_ uptake capacities for the azo-linked systems typically fall within a range from 20 to 35 mg g^−1^, showing good qualitative agreement with experimentally measured values despite the amorphous nature of the real materials. A key outcome of the computational analysis was that variations in linker length and connector substitution had only a modest impact on CO_2_ uptake within the azo-linked series. Extending the distance between trigonal nodes or introducing additional phenyl spacers failed to yield a systematic increase in adsorption capacity [63].

Further computational analysis focused on frameworks constructed from tetragonal tetraphenylporphyrin building blocks, either directly connected through azo bonds or separated by linear, bent, or trigonal linkers [33]. In these systems, the porphyrin unit itself plays an active role in CO_2_ binding, serving as an additional adsorption site alongside the azo linkage. Periodic DFT and GCMC simulations revealed that such POPs can achieve moderate to relatively high CO_2_ uptakes, with adsorption performance strongly dependent on framework dimensionality, pore accessibility, and stacking arrangement. Systems featuring linker-bridged porphyrin units, including 2D layered and 3D pto-type networks, exhibited calculated CO_2_ capacities of up to 49 mg g^−1^, which is in good agreement with the experimentally measured values of approximately 38 mg g^−1^ for the most porous materials in the series. In contrast, frameworks composed of directly connected azo-linked porphyrin units exhibited significantly lower surface areas, yet maintained non-negligible CO_2_ uptakes (23 mg g^−1^), indicating that strong local interactions at the azo and porphyrin sites can partially compensate for the reduced porosity [33].

Most recently, the computational analysis was extended to a new set of azo-linked porphyrin POPs (APPs) in which the linker chemistry was tuned using heteroatom-containing (hydroxylated biphenyl and anthraquinone) versus sterically hindered (methylated biphenyl and methylated phenyl) diamines [34]. Although these polymers are amorphous, periodic DFT and GCMC simulations on ordered layered reference models reproduced the key qualitative trend seen experimentally—the hydroxylated derivative (APP-BP-OH) outperforms its methylated analogue in CO_2_ uptake despite a lower surface area. This confirms that adsorption is governed not only by porosity but also by the local chemical environment. Analysis of electrostatic potential maps and CO_2_ density distributions showed that adsorption is dominated by nitrogen-rich porphyrin/azo motifs, while hydroxyl and carbonyl groups introduce additional accessible binding regions that can further enhance uptake. Importantly, the study also explored alternative stacking arrangements and showed that small lateral shifts between layers can strongly increase pore accessibility and, consequently, the predicted CO_2_ uptake. For APP-BP-OH, the eclipsed AA model yields 25 mg g^−1^ (1750 m^2^ g^−1^), whereas a serrated AA′ offset significantly raises the calculated uptake to 213 mg g^−1^ (2716 m^2^ g^−1^), with the staggered AB model providing an intermediate 85 mg g^−1^ (1894 m^2^ g^−1^). In addition, uptake trends were complemented with zero-loading enthalpies (−13.6 to −14.9 kJ mol^−1^) and BSSE-corrected DFT interaction energies, showing stronger CO_2_ binding to hydroxyl than to carbonyl sites (−31 vs. −27 kJ mol^−1^), consistent with the slightly higher simulated uptake of APP-BP-OH (25 mg g^−1^) compared with APP-AQ (23 mg g^−1^). Overall, these results provide a clear mechanistic link between linker functionalization, stacking accessibility, and CO_2_ adsorption in azo-linked porphyrin POPs [34].

Analysis of CO_2_ density distributions and electrostatic potential maps showed that favorable adsorption occurs preferentially near the azo linkages and around the porphyrin rings, particularly when these sites are sterically accessible on both sides of the porphyrin plane, as in hypothetical AB stacking configurations. However, such arrangements were computationally found to be less stable than the eclipsed AA stacking. These findings are consistent with conclusions from trigonal azo-linked frameworks that, in azo-based POPs, CO_2_ adsorption is governed not only by the overall surface area but also by the local electrostatic landscape and the accessibility of the nitrogen-rich motifs. Despite the idealized nature of the models and the lower experimental BET surface areas of the synthesized amorphous polymers, the calculations provide useful qualitative guidance by revealing how stacking, accessibility, and local polarity affect adsorption trends rather than by reproducing the exact structure of real disordered solids. For example, the experimental CO_2_ uptake of 32 mg g^−1^ (at 303 K) for the pyridine-based azo-linked polymer (TPP-azo) closely matches the GCMC-predicted values for laterally displaced stacking models (31 mg g^−1^ for AB staggered and 37 mg g^−1^ for inclined AA′ configuration).

### 4.3. Computational Studies of Azo-Linked POPs Beyond CO_2_ Adsorption

An early example of a computational study related to gas adsorption in a porous material containing azo bonds was reported for a framework obtained by the reaction of 4,4′-azodianiline with 1,3,5-triformylphloroglucinol, in which the azo linkage is indirectly introduced into the framework through the azodianiline moiety [93]. Adsorption of CO_2_, N_2_, CH_4_, and H_2_ was investigated experimentally. However, the computational analysis was limited to GCMC simulations of H_2_ adsorption only, performed on an idealized eclipsed structural model. Interpretations of CO_2_ selectivity relied primarily on experimental trends and qualitative arguments related to the CO_2_-philicity and N_2_-phobicity of azo groups [94]. The same framework served as a model system in a study that employed a dispersion-corrected periodic DFT to demonstrate that the widely assumed eclipsed AA stacking is not an energy minimum. Instead, calculations revealed several low-energy slipped configurations. Beyond structural implications, the study shows that stacking displacement can have a strong effect on electronic structure (e.g., band gap) [95]. This conclusion was further strengthened by a finite-temperature study based on machine learning force fields trained on DFT data, which showed that eclipsed stacking is dynamically unstable under ambient conditions and that slipped configurations are entropically stabilized. Simulated PXRD patterns averaged over dynamic trajectories reproduced experimental diffraction features more accurately than static eclipsed models [96].

These findings are consistent with previous reports. Several computational and combined experimental-theoretical studies have systematically investigated interlayer stacking modes in 2D COFs and their influence on structural and electronic properties. Although performed on other systems, such as imine or boronate-based COFs, these works often demonstrate that fully eclipsed AA stacking is energetically less favorable, primarily due to interlayer π-π repulsion and electrostatic interactions between adjacent aromatic layers. Early molecular mechanics and DFT studies mapped the potential energy surfaces (PES) of dozens of COFs and showed that energy minima generally occur for horizontally displaced layers, with offsets of approximately 1–2 Å relative to the eclipsed geometry [97,98]. Subsequent diffraction-based analysis and simulations demonstrated that inclined (AA′) and zigzag (AB) stacking modes reproduce experimental PXRD patterns more accurately than eclipsed models, particularly for materials with limited crystallinity [99]. Experimental studies employing advanced techniques, such as solid-state NMR [100] further confirmed that the stacking disorder and polymorphism [101] are intrinsic features of layered COFs, rather than defects or minor perturbations.

In parallel, a purely computational study used periodic DFT to propose hypothetical azo-linked COFs constructed from benzene, triphenylamine, triphenylbenzene, and triphenyltriazine connectors as conjugated semiconductors, and focused on electronic properties relevant for photocatalysis. In this work, the azo bond was treated as a backbone linkage enabling extended conjugation, but the calculations focused on band structures and band gaps from single-layer periodic 2D sheets. Although no adsorption simulations were performed, the study demonstrated that azo-linked frameworks exhibit electronic properties distinct from those of more commonly studied imine-linked analogues [102].

Finally, a practical synthetic route to true azo-linked COFs was established via imine to azo linkage exchange [103]. DFT was primarily employed to rationalize the electronic consequences of introducing the –N=N– linkage into the backbone, most notably bandgaps change consistent with experimental photophysical and photocatalytic trends. The computational study assumed idealized eclipsed AA stacking and focused on electronic structure, without directly addressing CO_2_ adsorption thermodynamics or stacking disorder.

### 4.4. Computational Study of Azodioxy and Azoxy-Linked Frameworks for CO_2_ Adsorption

One motivation for examining oxygenated N–N linkages was the hypothesis that azodioxy-based materials might exhibit a higher degree of structural order than their azo analogues. The idea was supported by the reported formation of crystalline 3D diamondoid networks from polynitroso monomers, enabling their characterization by single-crystal X-ray diffraction [59]. Computational methods proved to be a valuable tool for guiding synthetic procedures, as they are relatively inexpensive and can be applied to a wide range of systems. In the initial computational screening, six idealized 2D POPs were constructed from trigonal benzene and triazine-based connectors linked through azo, azoxy, or azodioxy bonds.

Binding energy calculations on simple molecular fragments (B3LYP-D3(BJ)/def2-TZVP, BSSE-corrected) showed that introducing oxygen atoms into the N–N linkage systematically enhances the interaction with CO_2_ compared with azo units. Among the three linkage types, azodioxy fragments gave the most favorable CO_2_ binding energies of about −20.9 kJ mol^−1^, followed by azoxy (–17.7 kJ mol^−1^) and azo (–16.5 kJ mol^−1^). This trend reflects an increase in local adsorption affinity at the linkage level [50]. However, its manifestation in bulk adsorption experiments depends on whether these high-affinity sites remain accessible within a sufficiently open and stable porous network.

Periodic DFT (PBE-D3/pob-TZVP-rev2) was used to optimize energetically preferred AA (eclipsed) and the less stable AB (staggered) configurations of the corresponding 2D POPs, followed by GCMC simulations of CO_2_/N_2_ adsorption at 298 K and 1 bar. Azoxy and especially azodioxy linkages create highly negative ESP regions along the pore walls, which correlate with localized CO_2_ density near these linkages and significantly enhanced CO_2_ uptake compared with azo analogues. In benzene-based frameworks, both AA and AB azodioxy-linked systems exhibit the highest calculated CO_2_ capacity overall, reaching 30 mg g^−1^ and 118 mg g^−1^, respectively. In contrast, triazine-based analogues reveal a more balanced behavior: the more planar azo-linked triazine structure AB can outperform its azodioxy counterpart, as layer distortion in the latter reduces the accessible adsorption sites. The study also highlighted the importance of slightly slipped stacking modes, such as AA′, where imperfect alignment of adjacent layers exposes additional high-affinity binding regions on both the linkages and the building blocks, further influencing the overall CO_2_ adsorption, from 31 mg g^−1^ for AA to 118 mg g^−1^ for AB. This observation is consistent with previously reported studies of boronate-based 2D COFs, where small lateral offsets between layers were energetically preferred and structurally more realistic than ideal AA packings [97,104].

This computational study was later extended to six additional POPs assembled from triphenylamine and triphenylpyridine building blocks linked through azo, azoxy, or azodioxy bonds. In this work, not only AA and AB but also inclined AA′ and serrated AA′ stacking modes were examined [51]. Modest lateral offsets between layers can enhance adsorption performance by exposing high-affinity regions on the framework that are partially shielded in the case of perfectly eclipsed AA geometry. For triphenylamine-based azodioxy systems, AB packings yielded the highest simulated CO_2_ uptakes of 177 mg g^−1^. In contrast, triphenylpyridine-based frameworks exhibited small energy differences between AA and AA′, suggesting that multiple stacking arrangements may coexist and jointly influence their CO_2_ adsorption behavior.

A central outcome of the studies was the clear correlation between the most negative ESP regions on the framework, located either on the azo nitrogen atoms (Figure 5c) or, more likely, on azoxy/azodioxy oxygen atoms (Figure 5d), and both the magnitude of CO_2_ uptake and the spatial distribution of CO_2_ density within the pores. This supports the use of ESP as a simple and computationally relatively inexpensive descriptor for pre-screening of candidate linkages and connectors before undertaking full GCMC simulations and offering at least a qualitative indication of the expected CO_2_ adsorption capacity.

To validate the stacking models, we combined periodic DFT and PXRD simulations for azodioxy polymers derived from polynitroso compounds with triphenyl-substituted benzene, pyridine, triazine, and amine cores. Four stacking arrangements (AA, inclined AA′, serrated AA′, and AB) were optimized using PBE-D3/pob-TZVP-rev2 level of theory and employed to simulate PXRD patterns. Comparison with experimental data revealed that the PXRD of the triphenyltriazine-based azodioxy polymer is best matched by a serrated AA′ stacking, and not by the simple eclipsed AA geometry that is commonly assumed for 2D COFs (Figure 3b). These observations are consistent with earlier computational studies suggesting that slipped configurations [50,51] can be energetically competitive and structurally realistic [50,51]. Such layer offsets can enhance CO_2_ uptake by exposing additional adsorption sites, providing a possible explanation for the adsorption behavior found in amorphous or partially ordered frameworks.

The computational models discussed in this section primarily compare azo, azoxy, and azodioxy linkages within idealized frameworks of related topology and accessibility in order to isolate the effects of linkage oxidation state, electrostatic environment, and stacking arrangement on adsorption behavior. Within these models, azoxy and especially azodioxy linkages generate more negative electrostatic regions and stronger local interactions with CO_2_ than their azo analogues. The experimentally realized azoxy/azodioxy-containing materials reported so far, however, generally exhibit low accessible surface area, limited long-range order, partial oligomerization, or poorly developed porosity, all of which can suppress the measured adsorption capacity. Thus, enhanced local CO_2_ binding in an idealized framework does not necessarily translate into high bulk CO_2_ uptake when the porous architecture of the real material remains insufficiently accessible.

## 5. Conclusions and Perspectives

Overall, this review highlights azo-linked POPs as a versatile platform for CO_2_ capture, where performance is governed by the interplay of building block topology and functionalization, porosity, and the local binding site environment. Across reported systems, enhanced CO_2_ adsorption can be achieved, yet uptake does not scale uniformly with BET surface area—most notably in porphyrin-based networks, where nitrogen-rich motifs and linker polarity make substantial contributions to CO_2_ affinity. Combined experimental and computational analysis further shows that pore accessibility and layer stacking can dominate adsorption trends—laterally displaced stackings often reproduce experimental uptakes better than ideal eclipsed models, particularly when several stacking arrangements are close in energy. Electrostatic descriptors (e.g., ESP) provide a practical route to locate high-affinity regions and rationalize CO_2_ density distributions.

The review also indicates, based on computational analysis, that oxygenated N–N linkages (azoxy/azodioxy) are a promising polarity-tuning handle that can strengthen CO_2_ binding in idealized frameworks. However, the few experimentally realized azoxy-/azodioxy-containing materials reported so far generally exhibit limited accessible porosity and structural order, which likely prevents this favorable linkage-level chemistry from being translated into high-bulk CO_2_ uptake. Reversible nitroso/azodioxide chemistry, therefore, remains a promising direction, particularly if the synthetic obstacles to obtaining sufficiently ordered and porous azodioxy-based networks can be overcome.

Several future directions emerge directly from the studies discussed in this review. A priority is the preparation of directly comparable azo-, azoxy-, and azodioxy-linked materials built from the same connector topology, so that the effect of linkage oxidation state on porosity, binding affinity, and selectivity can be established experimentally within structurally matched systems. Another priority is the development of synthetic and activation strategies that preserve permanent porosity in oxygenated N–N-linked networks, particularly azodioxy-based systems, where the favorable local CO_2_ binding predicted by computation has not yet been fully realized at the materials level. It would also be valuable to explore linkage-conversion strategies such as the imine-to-azo transformation demonstrated in COFs, as such approaches may provide a route to combining preorganized framework architectures with the enhanced local polarity of oxygenated N–N linkages. Equally important is the closer integration of computation with experiment, including validation of predicted adsorption sites and stacking models through adsorption enthalpies, mixed-gas measurements, and PXRD or total-scattering analyses.

Although substantial progress has been made, the translation of POP adsorbents—particularly N–N-linked variants—to industrial CO_2_ capture remains constrained by material stability, production costs, and scalability. Moving forward, assessing their practical applicability will require shifting focus from equilibrium uptake under dry, single-gas conditions toward a more realistic evaluation of adsorption kinetics, working capacity under relevant pressure or temperature conditions, and long-term cycling durability. Specifically, future research should prioritize: (i) enhancing stability under humidity and complex gas mixtures; (ii) reducing synthesis costs and time through greener, solvent-free, or energy-efficient protocols; and (iii) improving process practicality, including material shaping and compatibility with existing infrastructure. In parallel, materials design should continue to leverage classical performance levers—such as pore volume and surface area—while optimizing framework polarity and functional site chemistry to further boost CO_2_ affinity and selectivity.

In addition, the development of multifunctional porous materials that combine capture with post-capture conversion (e.g., by incorporating catalytic sites for photo- or electro-driven utilization) without compromising adsorption performance represents another promising direction. Azo-linked POPs with additional nitrogen-rich motifs (e.g., porphyrins, triazines, and pyridines) are especially attractive as heterogeneous catalysts for CO_2_ conversion into high-value chemicals, such as cyclic carbonates. Furthermore, porphyrin-based POPs allow for incorporating metal ions to enhance their catalytic performance. Another is the move toward composite and hybrid architectures, where combining different adsorbents can improve robustness, moisture tolerance, and regeneration efficiency under application-specific conditions. Finally, computational chemistry and machine learning are playing an increasingly important role—simulations can screen functional motifs and pore environments, estimate adsorption and regeneration-relevant descriptors, and help rationalize stability and performance trends; expanding, well-curated experimental/computational datasets will further improve machine learning predictiveness and accelerate optimization processes. Overall, advancing POPs adsorbents will require tighter coupling of synthetic innovation, stability/regeneration testing, and computation-guided design, supported by cross-disciplinary collaboration and practical and economic considerations.

## Figures and Tables

**Table 1:** Calculated average surface areas and CO_2_ uptakes (at 298 K and 1 bar). Adapted from [33,34,50,51,63].

Compound	Average Surface Area (m^2^ g^−1^)	CO_2_ Uptake (mg g^−1^)
TPB-azo (AA) ^a^	1957	19
TPB-azo (AB) ^a^	2462	65
TPB-azoxy (AA) ^a^	1830	28
TPB-azoxy (AB) ^a^	2284	73
TPB-azodioxy (AA) ^a^	1691	31
TPB-azodioxy (AB) ^a^	1949	118
TPT-azo (AA) ^a^	1828	14
TPT-azo (AB) ^a^	2411	92
TPT-azoxy (AA) ^a^	1704	19
TPT-azoxy (AB) ^a^	2077	73
TPT-azodioxy (AA) ^a^	1596	21
TPT-azodioxy (AB) ^a^	1635	44
TPA-azo (AA) ^b^	1858	23
TPA-azo (AB) ^b^	2327	153
TPA-azo (AA’_incl_) ^b^	1395	46
TPA-azo (AA’_serr_) ^b^	1911	28
TPA-azoxy (AA) ^b^	1686	38
TPA-azoxy (AB) ^b^	2051	169
TPA-azodioxy (AA) ^b^	1501	48
TPA-azodioxy (AB) ^b^	1570	177
TPP-azo (AA) ^b^	1880	18
TPP-azo (AB) ^b^	1894	31
TPP-azo (AA’_incl_) ^b^	132	37
TPP-azo (AA’_serr_) ^b^	1783	18
TPP-azoxy (AA) ^b^	1588	21
TPP-azoxy (AA’_incl_) ^b^	194	37
TPP-azodioxy (AA) ^b^	1636	27
TPP-azodioxy (AA’_incl_) ^b^	342	68
AZO-B ^c^	1957	20
AZO-B-PPD ^c^	2237	19
AZO-B-BZD ^c^	2306	19
AZO-T ^c^	1828	14
AZO-T-PPD ^c^	2127	14
AZO-T-BZD ^c^	2207	15
APP-1 ^d^	1969	13
APP-2 ^d^	2246	18
APP-3 ^d^	1931	15
APP-4 ^d^	1934	20
APP-5 ^d^	6777	49
APP-6 ^d^	7567	49
APP-7 ^d^	1332	23
APP-8 ^d^	1332	23
APP-BP-OH (AA) ^e^	1750	25
APP-BP-OH (AB) ^e^	2716	85
APP-BP-OH (AA’_serr_) ^e^	1894	213
APP-AQ ^e^	1699	23
APP-BP-Me ^e^	1805	20
APP-Ph-Me ^e^	1660	23

^a^ [50]; ^b^ [51]; ^c^ [63]; ^d^ [33]; ^e^ [34].

## Data Availability

No new data were created or analyzed in this study. Data sharing is not applicable to this article.
